# Plasticity in Meristem Allocation as an Adaptive Strategy of a Desert Shrub under Contrasting Environments

**DOI:** 10.3389/fpls.2017.01933

**Published:** 2017-11-09

**Authors:** Weiwei She, Yuxuan Bai, Yuqing Zhang, Shugao Qin, Zhen Liu, Bin Wu

**Affiliations:** ^1^Yanchi Research Station, School of Soil and Water Conservation, Beijing Forestry University, Beijing, China; ^2^Key Laboratory of State Forestry Administration on Soil and Water Conservation, Beijing Forestry University, Beijing, China; ^3^Engineering Research Center of Forestry Ecological Engineering, Ministry of Education, Beijing Forestry University, Beijing, China

**Keywords:** *Artemisia ordosica*, biomass allocation, desert shrub, life-history strategy, meristem fate, reproductive allocation, stressful environment

## Abstract

The pattern of resource allocation to reproduction vs. vegetative growth is a core component of a plant’s life-history strategy. Plants can modify their biomass allocation patterns to adapt to contrasting environments. Meristems can have alternative fates to commit to vegetative growth, reproduction, or remaining inactive (dormant or senescent/dead). However, knowledge about whether meristem fates can interpret adaptive changes in biomass allocation remains largely unknown. We measured aboveground plant biomass (a proxy of plant size) and meristem number of a dominant shrub *Artemisia ordosica* in three populations occupying different habitats in the Mu Us Desert of northern China. Size-dependent biomass allocation and meristem allocation among habitats were compared. The size-dependent biomass allocation and meristem allocation of *A. ordosica* strongly varied across habitats. There were significant positive linear relationships between meristem allocation and biomass allocation in all habitats, indicating that meristem allocation is an indicator of the estimated resource allocation to reproductive and vegetative organs in this species. Plasticity in meristem allocation was more likely caused by larger individuals having less active meristems due to environmental stress. Vegetative meristems (VM) were likely more vulnerable to environmental limitation than reproductive ones, resulting in the ratio of resource investment between vegetative and reproductive functions exhibiting plasticity in different habitats. *A. ordosica* invested a higher fraction of its resource to reproduction in the adverse habitat, while more resource to vegetative growth in the favorable habitat. *A. ordosica* adopts different resource allocation patterns to adapt to contrasting habitat conditions through altering its meristem fates. Our results suggest that the arid-adapted shrub *A. ordosica* deactivates more VM than reproductive ones to hedge against environmental stress, representing an important adaptive strategy. This information contributes to understand the life-history strategies of long-lived plants under stressful environments.

## Introduction

Growth and reproduction are two of the most fundamental life-history functions in plants. The total amount of available resources at a given time is limited; therefore, once resources are allocated to a particular function, they are not available for other functions, resulting in a consequential investment trade-off between life-history functions (Stearns, 1989; Obeso, 2002; Reekie and Avila-Sakar, 2005). The pattern of resource (biomass) allocation to reproduction vs. vegetative growth is a central issue in plant life-history studies (Bonser and Aarssen, 2009; Weiner et al., 2009a). Different patterns of resource allocation usually reflect plant’s adaptive strategies that are the product of natural selection pressures and constraints (Bonser and Aarssen, 1996; Weiner, 2004). Plants can modify their allocation patterns to maximize their overall fitness in different environments (Guo et al., 2012). Therefore, studies of plant allocation patterns under different conditions could provide insights into our understanding of the adaptive strategies of plants to different environments.

A major question is how environmental conditions affect plant allocation patterns. Plant allocation patterns are almost allometric, i.e., size-dependent (Weiner, 2004; Xie et al., 2015). Consequently, any factor that affects plant size will also influence resource allocation pattern. In some cases, environmental factors only influence plant size, but not the pattern of resource allocation (Zhang et al., 2008; Weiner et al., 2009b). In other cases, both plant size and allocation pattern are affected (Guo et al., 2012; Tian et al., 2012). Many studies of annual and biennial plants have shown that resource allocation is mainly limited by plant size (Weiner et al., 2009a,b). In comparison, studies have shown that perennial plants exhibit more plasticity in their resource investment in relation to different environmental conditions (Weiner et al., 2009a; Guo et al., 2012; Delerue et al., 2013; Santos-del-Blanco et al., 2013). For instance, under favorable environments, perennial plants may invest more resource to reproduction at larger individuals due to a low risk to longer-term survival (Guo et al., 2012; Delerue et al., 2013). However, under adverse conditions, perennial plants may reduce resource allocation to reproduction with increasing plant size. This response might be an adaptive strategy to increase longer-term survival (Delerue et al., 2013) or might be the consequence of environmental limitation on larger individuals (e.g., there might be more damage to buds and meristems in larger individuals) (Salguero-Gómez and Casper, 2011; Guo et al., 2012).

This issue raises the question of what mechanisms underlie the plasticity of resource allocation in perennial plants. One possible explanation is that the plasticity of resource allocation might be strongly correlated with the conversion efficiency of resource from vegetative tissue to reproduction (Weiner et al., 2009a; Guo et al., 2012). For example, plants living under unfavorable environments might show lower conversion efficiency of biomass to reproduction at larger individuals (i.e., the cost of maintaining reproductive structures increases as plants grow larger) (Guo et al., 2012). Alternatively, resource allocation could be measured in terms of the allocation of meristems, the original tissues from which vegetative and reproductive structures arise (Geber, 1990; Bonser and Aarssen, 2001; Lehtilä and Larsson, 2005). Meristems can have alternative fates to commit to vegetative growth, reproduction, or remaining inactive (dormant or senescent/dead), suggesting that the plasticity of resource allocation to reproduction vs. growth is possible (Watson, 1984; Lovett Doust, 1989; Weiner et al., 2009a). However, existing results from herbaceous plants have not found evidence for plasticity in meristem allocation to reproductive vs. vegetative functions (Lehtilä and Larsson, 2005; Zhang et al., 2008; Weiner et al., 2009a).

To examine how environmental conditions influence biomass allocation and meristem allocation in perennial plants, we investigated individuals of a dominant woody shrub (*Artemisia ordosica* Krasch) population across three distinct dune habitats [fixed dunes covered with biological soil crusts (FC), fixed dunes (FD), and semi-fixed dunes (SF)] in the Mu Us Desert, northern China. The three study habitats exhibit different growth conditions for plants, with FC exhibiting higher vegetation coverage and resource retention capacity than SF (Kobayashi et al., 1995; Jin et al., 2015). A previous study of *A. ordosica* showed that SF support much higher population growth and plant fecundity than the other two habitats, suggesting this species adopts different resource allocation patterns across these different dune habitats (Li et al., 2011). Specifically, we address the following questions: (i) whether meristem allocation can be used as a surrogate for estimating resource allocation in *A. ordosica*; (ii) how habitat conditions affect the patterns of biomass and meristem allocation; and (iii) whether meristem fates can interpret the adaptive changes in resource allocation of *A. ordosica* across different habitats.

## Materials and Methods

### Study Species and Area

*Artemisia ordosica* Krasch (Asteraceae) is a long-lived, deciduous, dwarf shrub that grows up to 100 cm in height. Its tap roots can reach 1–3 m deep, while the lateral roots are mainly distributed in the upper soil layer (0–30 cm) (Li et al., 2010). Adult individuals have brown older branches and purple current-year twigs (Supplementary Figure S1). Current-year twigs sprout from old branches and consist of vegetative twigs (VT) and reproductive twigs (RT). RT flower in June and set seed in August. The seeds mature in October, and the twigs die in winter. VT can survive the winter and generate new VT or RT the following spring (She et al., 2015). The leaves of VT are the fundamental photosynthetic organs of individual plants. In comparison, RT are inherently photosynthate sinks. Recruitment of new plant individuals is almost exclusively from seeds (Li et al., 2011).

This study was conducted at Yanchi Research Station (37°04′–38°10′N, 106°30′–107°41′E, 1530 m a.s.l), which is located on the southwestern edge of the Mu Us Desert, Ningxia, China. This region has a semiarid continental monsoon climate with an average annual temperature of 8.1°C and a mean annual precipitation of 284.8 mm (1955–2013) (She et al., 2016). Soil type is characterized by quartisamment based on the US Soil Taxonomy (Gao et al., 2014). The landscape of this region is typical inland dune ecosystems with very distinct habitat types, including FD, SF, and mobile dunes. Dune type is empirically defined by vegetation coverage (FD, ≥30%; SF, 10 ∼ 30%; mobile dunes, <10%) (Yan and Cong, 2015). In well-developed FD (vegetation coverage >50%), the ground surface is largely covered by biological soil crusts (Li et al., 2011). The fine soil component, resource retention capacity and wind erosion generally decline from well-developed FD to mobile dunes (Kobayashi et al., 1995; Jin et al., 2015; Zhu, 2015). Vegetation coverage is generally used as a surrogate for habitat condition in this ecosystem type (Kobayashi et al., 1995; Wei et al., 2016). *A. ordosica* can dominate vast dune habitats, except for mobile dunes (Kobayashi et al., 1995).

### Study Design and Data Collection

Three dune habitats were selected for this study; i.e., FC, FD, and SF (Supplementary Figure S2). These habitats were located at a distance of 2–4 km from each other. The overall plant cover was about 60, 30, and 10% in FC, FD, and SF habitat, respectively. Our previous studies show that the SF habitat are characterized by a higher fraction of coarse texture (Zhu, 2015), lower nutrient availability (Zhu, 2015), and more wind erosion (Wang et al., 2008) when compared to the FC habitat. At the end of the growing season (late August, 2014), three plots were established in each habitat. Plots measured 15 m × 15 m in FC and FD habitats and 20 m × 20 m in SF habitat, where plant density was quite low. To cope with the practical challenges of meristem counting and biomass harvest, we used a stratified systematic sampling approach to collect our data. Specifically, we measured the dimensions of plant crowns [maximum crown width (C_1_) and minimum crown width (C_2_)] in all plots. Small plants (generally shorter than 20 cm) without fully developed canopy were excluded from our measurements. Shrub size distribution was estimated from canopy area (CA) as: CA = π × C_1_/2 × C_2_/2. In each habitat, we chose 31 individuals, representing a wide range of sizes, for data collection using stratified systematic sampling based on the shrub size distribution (three levels with: CA < 0.5 m^2^, 0.5 < CA < 1.0 m^2^, CA > 1.0 m^2^).

For the small individuals (CA < 0.5 m^2^), the number of VT and RT was counted in the whole plant canopy. For the intermedium (0.5 < CA < 1.0 m^2^) and large individuals (CA > 1.0 m^2^), we counted the number of VT and RT from one half and one quarter of the canopy, respectively. The number of vegetative meristems (VM) and reproductive meristems (RM) was equivalent to the number of VT and RT, respectively. Active meristem (AM) number was the sum of the number of VM and RM, i.e., the number of current-year twigs (CYT). We randomly measured the length of ∼10 twigs of each type per individual. Subsequently, the selected individuals were harvested above ground, and were separated into VT, RT, and woody parts (containing dead branches). All plant samples were oven-dried at 70°C for 48 h and weighed. The reproductive and vegetative output of each plant were estimated as the biomass of RT and VT, respectively. Plant size was determined by the above ground biomass of each individual.

### Data Analyses

We analyzed size-dependent meristem allocation and biomass allocation, in addition to the relationships between meristem allocation and biomass allocation, using the classical allometric model: log *Y* = *b* + *a* log *X*, where *X* and *Y* are two plant traits, and parameters *a* and *b* are the scaling slope and the allometric intercept, respectively. Allometric analysis, a method for testing and interpreting allometric relationships (e.g., reproductive output vs. plant size), is useful for distinguishing the size-dependent and size-independent effects on variation in plant biomass allocation (Poorter and Sack, 2012; Tian et al., 2012; Wang et al., 2016). According to the allometric perspective, only changes in the allometric relationship of a genotype in different environments are regarded as modifications to the pattern of allocation (i.e., plasticity in allocation exists) (Weiner et al., 2009a).

Standardized major axis (SMA) regression was used to fit our allometric data and determine the parameters. We tested whether the slope among individuals of each habitat differed from 1, and whether there were significant differences in slopes among habitats. To determine the relationships between meristem allocation and biomass allocation, we analyzed the RT biomass–RM number relationship, the VT biomass–VM number relationship and the relationship between the RT:VT biomass ratio and the RM:VM number ratio in the three different habitats. To examine how habitat conditions affect the pattern of meristem and biomass allocation, we tested the relationships between plant biomass and twig biomass (CYT, RT, and VT), meristem number (AM, RM, and VM) and average twig length (CYT, RT, and VT) across the three habitats. We also compared the slopes of size-dependent biomass allocation with those of size-dependent meristem allocation among habitats. Linear or binominal regression was used to determine the relationship between plant biomass and the RM:VM number ratio in the three different habitats.

All data were log-transformed to meet the assumptions of normality prior to statistical analyses. Statistical significance was determined at a level of *P* ≤ 0.05. All analyses and figures were completed using R version 3.3.1 (R Core Team, 2016). We used the package SMATR 3 (Warton et al., 2012) for SMA regressions and ggplot2 (Wickham, 2009) for constructing graphs.

## Results

**FIGURE 1:**
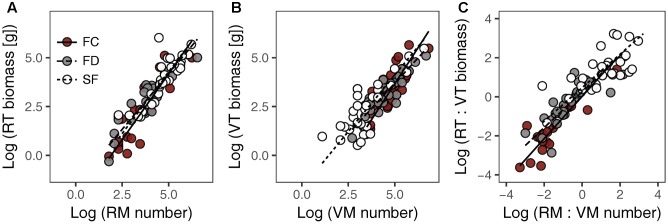
Relationships between meristem allocation and resource allocation to reproductive and vegetative parts of *Artemisia ordosica* across habitats: **(A)** RM number and RT biomass, **(B)** VM number and VT biomass, **(C)** RM:VM number and RT:VT biomass. FC, fixed dunes with crusts; FD, fixed dunes; SF, semi-fixed dunes; RT, reproductive twig; VT, vegetative twig; RM, reproductive meristem; VM, vegetative meristem.

There were significant positive relationships between RT biomass and RM number (RT–RM), VT biomass and VM number (VT–VM) and the RT:VT biomass ratio and the RM:VM number ratio (RT:VT–RM:VM) in all habitats (**Figure 1** and **Table 1**). Habitat conditions had no significant effect on the slope of the RT–RM relationship or that of the RT:VT–RM:VM relationship; however, it did affect the VT–VM slope. The *R*^2^ values of the RT–RM relationship, the VT–VM relationship and the RT:VT–RM:VM relationship among habitats fell within the ranges 0.72–0.84, 0.67–0.73, and 0.56–0.80, respectively. The overall *R*^2^ value of the RT:VT–RM:VM relationship was 0.80, and its slope was not significantly different from 1 (*P* = 0.598).

**Table 1:** Estimated slopes and intercepts of allometric relationships between meristem allocation and resource allocation to the reproductive and vegetative parts of *Artemisia ordosica* across habitats.

	Habitat	*n*	*R*^2^	*P*	Slope	Intercept	95% CIs of slope	95% CIs of intercept	H0: slope = 1	H0: slopes are equal
									*P*	*P*
Log(RT biomass) vs.	FC	22	0.81	<0.001	1.35 a	-2.58	(1.10, 1.65)	(-3.66, 1.50)	0.005	0.407
log(RM number)	FD	27	0.84	<0.001	1.14 a	-1.53	(0.97, 1.34)	(-2.29, -0.76)	0.107	
	SF	31	0.72	<0.001	1.26 a	-2.05	(1.04, 1.54)	(-3.24, -0.85)	0.022	

Log(VT biomass) vs.	FC	31	0.70	<0.001	1.47 a	-3.69	(1.20, 1.81)	(-5.19, -2.20)	<0.001	0.047
log(VM number)	FD	31	0.73	<0.001	1.03 b	-1.48	(0.85, 1.26)	(-2.42, -0.54)	0.730	
	SF	31	0.67	<0.001	1.16 ab	-1.68	(0.93, 1.44)	(-2.61, -0.75)	0.172	

Log(RT:VT biomass) vs.	FC	22	0.80	<0.001	1.08 a	0.02	(0.88, 1.33)	(-0.41, 0.44)	0.448	0.315
log(RM:VM number)	FD	27	0.66	<0.001	0.92 a	0.26	(0.72, 1.17)	(-0.04, 0.55)	0.479	
	SF	31	0.56	<0.001	0.85 a	0.47	(0.67, 1.10)	(0.08, 0.85)	0.212	

*Values with different letters are significantly different at *P* ≤ 0.05. FC, fixed dunes with crusts; FD, fixed dunes; SF, semi-fixed dunes; RT, reproductive twig; VT, vegetative twig; RM, reproductive meristem; VM, vegetative meristem*.

**FIGURE 2:**
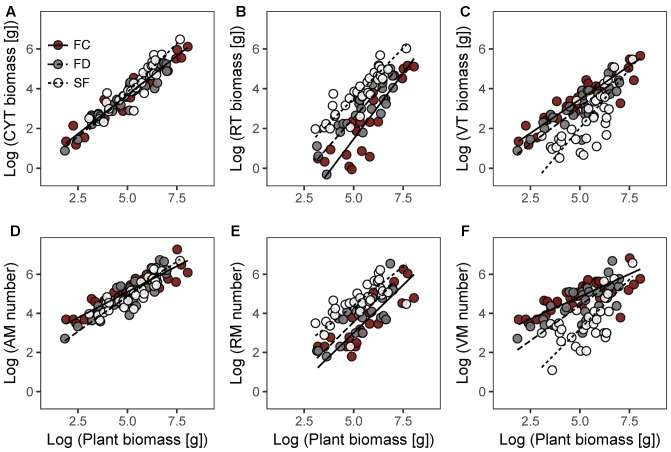
Allometric relationships between plant biomass and CYT biomass **(A)**, RT biomass **(B)**, VT biomass **(C)**, AM number **(D)**, RM number **(E)**, VM number **(F)** of *Artemisia ordosica* in three dune habitats. FC, fixed dunes with crusts; FD, fixed dunes; SF, semi-fixed dunes; CYT, current-year twig; RT, reproductive twig; VT, vegetative twig; AM, active meristem; RM, reproductive meristem; VM, vegetative meristem.

In each habitat, there were highly significant relationships between CYT biomass and plant biomass (CYT–P), RT biomass and plant biomass (RT–P), VT biomass and plant biomass (VT–P), AM number and plant biomass (AM–P), RM number and plant biomass (RM–P), and VM number and plant biomass (VM–P) (**Figure 2** and **Table 2**). Habitat conditions had no significant effect on the RT–P slope and the RM–P slope, but it did significantly affect the slopes of CYT–P, VT–P, AM–P, and VM–P. The slopes of CYT–P and VT–P in SF habitat were significantly higher than those in FC and FD habitats. The slopes of AM–P and VM–P in FC habitat were significantly lower than those in FD and SF habitats. In all habitats, the slopes of RT–P and RM–P were generally not significantly different from 1. The AM–P slope in all habitats was significantly lower than 1. The slopes of CYT–P, VT–P, and VM–P in FC and FD habitats were significantly lower than 1, and those slopes in SF habitat were no different from 1. In all habitats, the slopes of the relationships between twig biomass (CYT, RT, and VT) and plant biomass were generally higher than those of the relationships between meristem number (AM, RM, and VM) and plant biomass (Supplementary Figure S3). FC habitat showed significant differences for these parameters.

**Table 2:** Estimated slopes and intercepts of allometric relationships between plant biomass and twig biomass, meristem number and twig length of *Artemisia ordosica* across habitats.

	Habitat	*n*	*R*^2^	*P*	Slope	Intercept	95% CIs of slope	95% CIs of intercept	H0: slope = 1	H0: slopes are equal
									*P*	*P*
Log(CYT biomass) vs.	FC	31	0.94	<0.001	0.78 a	-0.23	(0.71, 0.86)	(-0.62, 0.15)	<0.001	0.039
log(plant biomass)	FD	31	0.91	<0.001	0.82 a	-0.44	(0.72, 0.91)	(-0.93, 0.04)	<0.001	
	SF	31	0.86	<0.001	0.98 b	-1.05	(0.85, 1.12)	(-1.80, -0.30)	0.721	
Log(RT biomass) vs.	FC	22	0.77	<0.001	1.32 a	-5.15	(1.06, 1.65)	(-6.87, -3.43)	0.015	0.150
log(plant biomass)	FD	27	0.71	<0.001	1.13 a	-3.13	(0.91, 1.41)	(-4.51, -1.75)	0.258	
	SF	31	0.80	<0.001	1.01 a	-1.54	(0.86, 1.19)	(-2.46, -0.62)	0.899	
Log(VT biomass) vs.	FC	31	0.84	<0.001	0.68 a	0.07	(0.58, 0.79)	(-0.47, 0.61)	<0.001	0.002
log(plant biomass)	FD	31	0.69	<0.001	0.74 a	-0.64	(0.60, 0.92)	(-1.48, 0.19)	0.007	
	SF	31	0.55	<0.001	1.18 b	-3.85	(0.91, 1.51)	(-5.49, -2.22)	0.203	
Log(AM number) vs.	FC	31	0.82	<0.001	0.53 a	2.46	(0.45, 0.62)	(2.01, 2.91)	<0.001	0.010
log(plant biomass)	FD	31	0.78	<0.001	0.73 b	1.26	(0.61, 0.86)	(0.58, 1.94)	<0.001	
	SF	31	0.79	<0.001	0.72 b	1.26	(0.61, 0.85)	(0.59, 1.93)	<0.001	
Log(RM number) vs.	FC	22	0.68	<0.001	0.98 a	-1.90	(0.76, 1.27)	(-3.41, -0.39)	0.874	0.426
log(plant biomass)	FD	27	0.63	<0.001	0.99 a	-1.41	(0.77, 1.27)	(-2.78, -0.03)	0.943	
	SF	31	0.50	<0.001	0.80 a	0.40	(0.61, 1.04)	(-0.77, 1.57)	0.097	
Log(VM number) vs.	FC	31	0.54	<0.001	0.46 a	2.56	(0.36, 0.59)	(1.94, 3.18)	<0.001	< 0.001
log(plant biomass)	FD	31	0.46	<0.001	0.72 b	0.81	(0.54, 0.95)	(-0.26, 1.88)	0.020	
	SF	31	0.36	<0.001	1.01 b	-1.88	(0.75, 1.37)	(-3.56, -0.19)	0.924	
Log(CYT length) vs.	FC	31	0.17	0.020	0.21	2.03	(0.15, 0.30)	(1.63, 2.42)	<0.001	–
log(plant biomass)	FD	31	0.06	0.202	0.14	2.26	(0.10, 0.20)	(1.97, 2.54)	–	
	SF	31	0.09	0.095	0.26	1.64	(0.18, 0.37)	(1.12, 2.16)	<0.001	
Log(RT length) vs.	FC	22	0.22	0.028	0.20	1.79	(0.13, 0.29)	(1.31, 2.26)	<0.001	–
log(plant biomass)	FD	27	0.08	0.146	0.18	1.96	(0.12, 0.26)	(1.57, 2.34)	–	
	SF	31	0.01	0.616	0.23	1.82	(0.16, 0.34)	(1.34, 2.31)	–	
Log(VT length) vs.	FC	31	0.23	0.006	0.23	1.99	(0.17, 0.32)	(1.58, 2.39)	<0.001	–
log(plant biomass)	FD	31	0.02	0.418	0.16	2.16	(0.11, 0.24)	(1.83, 2.49)	–	
	SF	31	0.25	0.004	0.38	0.78	(0.28, 0.53)	(0.10, 1.47)	<0.001	

*Values with different letters are significantly different at *P* ≤ 0.05. For the cases with non-significant allometric relationships, slope comparisons among habitats were not performed. FC, fixed dunes with crusts; FD, fixed dunes; SF, semi-fixed dunes; CYT, current-year twig; RT, reproductive twig; VT, vegetative twig; AM, active meristem; RM, reproductive meristem; VM, vegetative meristem.*

The relationships between twig length and plant biomass varied among habitats and twig types (**Table 2**). In FC habitat, the average twig length of CYT, VT, and RT significantly and positively increased with plant biomass. In SF habitat, only CYT length and VT length were positively correlated with increasing plant biomass. In FD habitat, there were non-significant relationships between twig length and plant biomass.

**FIGURE 3:**
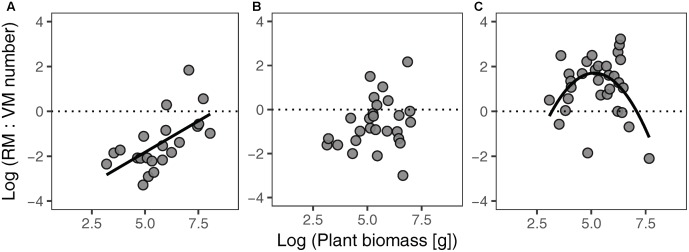
Relationships between the RM:VM number ratio and plant biomass of *Artemisia ordosica* across habitats for FC **(A)**, FD **(B)**, and SF **(C)**. FC: *y* = 0.56 *x* – 4.61, *P* = 0.002, *R*^2^ = 0.38; FD: *P* = 0.206, *R*^2^ = 0.06; SF: *y* = –0.45 *x*^2^ + 4.61 *x* – 10.13, *P* = 0.030, *R*^2^ = 0.22. FC, fixed dunes with crusts; FD, fixed dunes; SF, semi-fixed dunes; RM, reproductive meristem; VM, vegetative meristem.

Different habitat types showed different relationships between the RM:VM number ratio and plant biomass (**Figure 3**). The ratio of RM:VM number was significantly linearly and non-linearly related to plant biomass in FC and SF habitats, respectively; while no significant relationship was found in FD habitat. In SF habitat, the highest value of the RM:VM number ratio was present at intermediate levels of plant biomass.

## Discussion

### Relationship between Meristem Allocation and Biomass Allocation

Our study showed that there was a positive, linear relationship between meristem allocation and biomass allocation in *A. ordosica* population across all study habitats (**Figures 1A,B**), indicating that meristem number could be used as a surrogate for estimating biomass allocation in this species. Other studies of herbaceous plants also found that meristem allocation mirrors resource allocation when using carbon (biomass) as a currency (Bonser and Aarssen, 2003; Lehtilä and Larsson, 2005). The relationship between the RT:VT biomass ratio and the RM:VM number ratio was positive, with slope not significantly different from 1 (**Figure 1C** and **Table 1**), suggesting that resource investment per meristem is constant in this woody perennial, regardless of its different meristem types. Meristems are the primordial tissues for the development of vegetative and reproductive organs (Geber, 1990; Bonser and Aarssen, 2001). Therefore, the availability of VM/RM might constrain vegetative/reproductive output (Geber, 1990; Bonser and Aarssen, 1996). In contrast to using biomass in allocation measures, meristem counting is non-destructive, which is important for studies where it is not feasible to harvest biomass. However, meristem counting is difficult in large plants (e.g., woody plants). Thus, efficient sampling strategies are needed to estimate the number of meristems in large organisms. Furthermore, the relationship between meristem allocation and resource allocation might be species dependent (Lehtilä and Larsson, 2005). Thus, additional research is needed to examine this relationship in other woody plants.

### Effects of Habitat Conditions on Allometric Relationships

In inland dune systems, different dune types show very distinct habitat conditions. It is widely reported that habitat conditions are more harsh and stressful in SF than that in well-developed FD (Kobayashi et al., 1995; Li et al., 2011; Jin et al., 2015). Previous studies in our study area also indicate that the SF are characterized by a higher fraction of coarse texture (Zhu, 2015), lower nutrient availability (Zhu, 2015), and more wind erosion (Wang et al., 2008) when compared to the well-developed FD. It is hard to consider one environmental factor as the proxy of stress posed by different dune types. The difference of environmental stress among dun types is a comprehensive outcome of many environmental factors (e.g., soil texture, water and/or nutrient availability or wind erosion).

Our results showed that habitat conditions mainly influence the allocation patterns of AM and VM of *A. ordosica*, rather than the allocation patterns of RM (**Figures 2D–F** and **Table 2**). In all habitats, the slopes of the relationships between AM number and plant biomass were significantly less than 1 (**Table 2**), suggesting that the number of AM is less in larger individuals. In desert environments, larger individuals need more available resources; thus, their growth is strongly limited by environmental conditions (Salguero-Gómez and Casper, 2011). Larger individuals might reduce their mortality risk under stressful conditions by deactivating more meristems (e.g., making them dormant or senescent/dead) (Shefferson, 2009; Gillespie and Volaire, 2017).

In FC and FD habitats, the slopes of the relationship between VM number and plant biomass were significantly less than 1, whereas those of RM number were no different from 1 (**Table 2**). This result suggests that larger individuals have fewer active meristems in these two habitats because of VM being inactive. In contrast to RT, the VT of *A. ordosica* are fundamental organs for photosynthesis. Consequently, the growth of VT is probably more related to external environmental conditions than that of RT. In comparison, the development of RT probably depends on the amount of stored carbohydrate resources in plant. Therefore, VM are probably subject to more environmental stress than RM. In addition, we found that the slopes of size-dependent AM and VM allocation in FC habitat were significantly lower than those in FD habitat (**Table 2**). This result suggests that larger individuals in FC habitat have fewer AM and VM. Vegetation coverage is higher in FC habitat, which resulting in a more serious competition for resource with the larger individuals. Consequently, larger individuals in FC habitat are more likely to suffer more environmental stress than those in FD habitat.

In SF habitat, we found that the slope of the relationship between VM number and plant biomass was no different from 1, whereas that of RM number was marginally significant less than 1 (**Table 2**). This result might be explained by inactive VM being homogeneous in plants of all sizes, whereas RM suffer more damage in larger individuals in this habitat. Environmental conditions are more stressful in SF habitat compared to the other two habitats (Wang et al., 2008; Zhu, 2015). Consequently, both VM and RM in SF habitat are subject to more environmental stress. This explanation is supported by our findings that intermediate sized plants had the highest value of the RM:VM number ratio in SF habitat (**Figure 3C**). In addition, our results show that the slopes of size-dependent VM and RM allocation in SF habitat were no different from those in FD habitat (**Table 2**). We guess that the large variation of our field data might mask the statistical significance of slope comparison between these two habitats.

The RM:VM number ratio of *A. ordosica* clearly varied across the three habitats (**Figure 3**). The development of RT is intrinsically supported by VT; thus, the higher value of the RM:VM number ratio of an individual implies higher reproductive burden. The RM:VM number ratio in SF habitat was higher than that in the other two habitats, suggesting that individuals in the SF population exhibit higher reproductive output. This suggestion is supported by the results of a previous study on *A. ordosica* (Li et al., 2011).

The present study showed that the patterns of biomass allocation of *A. ordosica* across habitats are similar to those of meristem allocation (**Figure 2** and **Table 2**). The plasticity of biomass allocation largely results from changes in the allocation pattern of vegetative parts. The allometric slopes of size-dependent biomass allocation were generally higher than those of size-dependent meristem allocation in all three habitats, with significant differences being detected in FC habitat (Supplementary Figure S3). These results suggest that, regardless of meristem number, other factors contribute to increasing twig biomass. We found that the average twig length of *A. ordosica* increased with plant size in FC and SF habitats (**Table 2**), which implies that larger individuals could adjust to the adverse effects of meristem limitation on twig biomass by elongating twigs.

### Implications of the Plasticity of Meristem Allocation on Plant Adaption

Our results suggest that *A. ordosica* adjusts the fate of meristems, with plasticity in resource allocation to reproduction and growth, to adapt to contrasting growing conditions in desert dune systems. Desert environments are generally characterized by large temporal variation in resource supply due to highly variable precipitation and frequent drought periods (Jia et al., 2016). Thus, larger plants are more likely to suffer from environmental stress (Salguero-Gómez and Casper, 2011). Larger individuals might survive adverse conditions better by having more inactive meristems. However, such individuals could increase twig length to buffer the adverse effects of inactive meristems on plant growth. Compared to RM, the VM of *A. ordosica* are probably more vulnerable to stressful conditions, resulting in the plasticity of meristem allocation, as well as plasticity in resource investment to vegetative and reproductive organs in different environments. Under adverse conditions, *A. ordosica* might increase reproductive output at the cost of reduced survival, providing the chance to disperse its offspring to colonize new, perhaps, richer or safer habitats. In comparison, under favorable conditions, more resources might be allocated to vegetative structures to strengthen the competition ability of *A. ordosica*, allowing it to maintain dominance in the local community.

## Conclusions

The plasticity in meristem allocation of *A. ordosica* is probably caused by larger individuals having fewer active meristems in response to environmental limitation. VM might be more vulnerable to environmental limitation than RM, resulting in resource investment to vegetative and reproductive organs being plastic under different growing conditions. Our results suggest that *A. ordosica* deactivates more VM than reproductive ones to safeguard against environmental stress, representing an important life history strategy to adapt to arid environments. The adaptive changes in meristem allocation of *A. ordosica* across different habitats possibly explain its dominance in the vast dune area of Mu Us Desert.

## Author Contributions

WS, YZ, and SQ conceived the ideas and designed methodology. WS and ZL collected the data. WS and YB analyzed the data. WS led the writing of the manuscript. YB, YZ, SQ, and BW assisted with revising the draft manuscript. All authors approved this manuscript.

## Conflict of Interest Statement

The authors declare that the research was conducted in the absence of any commercial or financial relationships that could be construed as a potential conflict of interest.
