# Supplementary material for: Plasticity in Meristem Allocation as an Adaptive Strategy of a Desert Shrub under Contrasting Environments
Source: Front Plant Sci. 2017 Nov 9;8:1933. doi: 10.3389/fpls.2017.01933 (PMC5684672; doi:10.3389/fpls.2017.01933)
Supplement: Supplementary file 1 [file Data_Sheet_1.doc]

Supplementary Material

**Plasticity in meristem allocation as an adaptive strategy of a desert shrub under contrasting environments**

**Weiwei She, Yuxuan Bai, Yuqing Zhang^*^, Shugao Qin, Zhen Liu, Bin Wu**

*Corresponding author: [zhangyqbjfu@gmail.com](mailto:zhangyqbjfu@gmail.com)


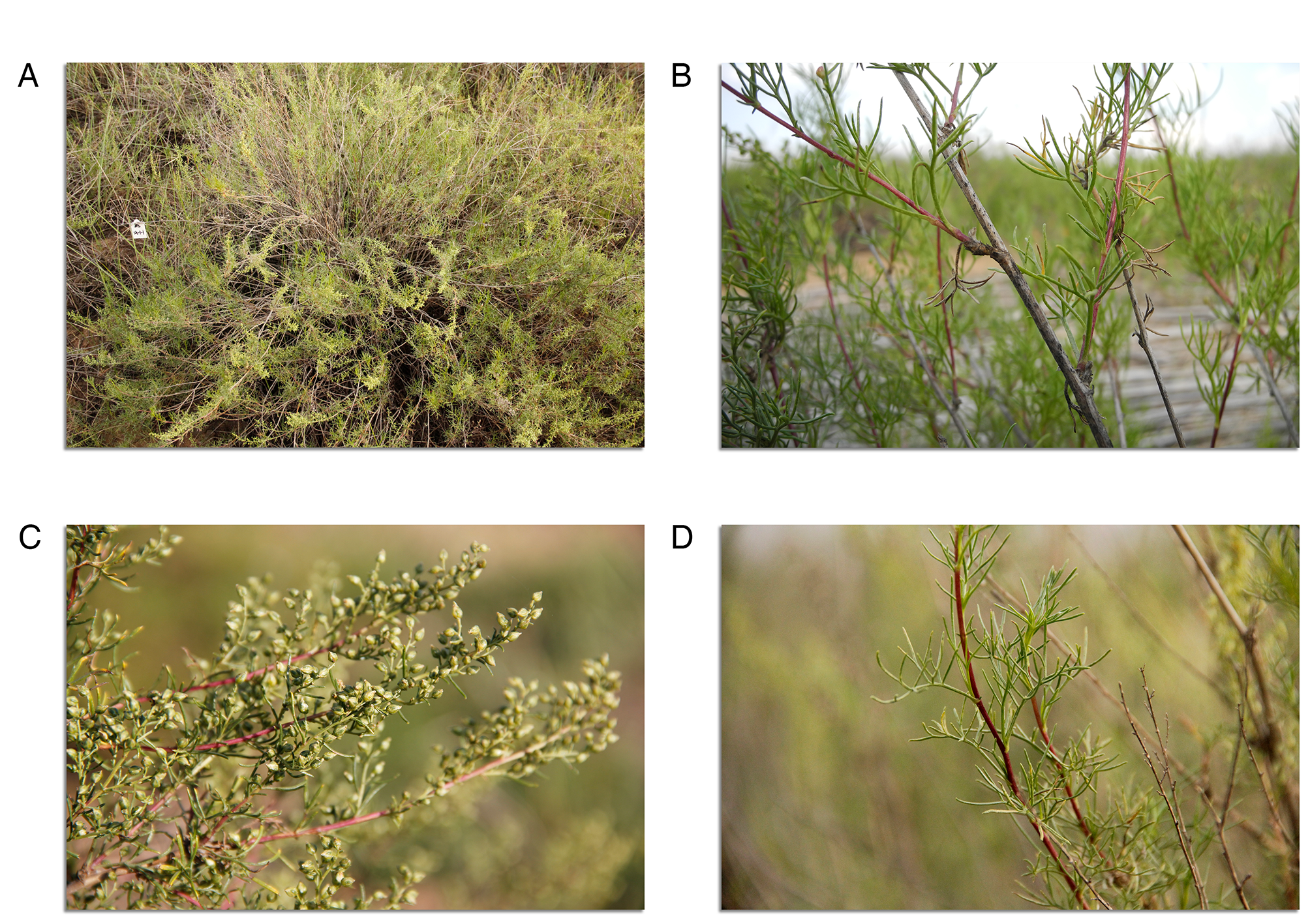


**Figure S1.** Photographs of *Artemisia ordosica*: (A) an adult individual, (B) branch systems with brown older branches and purple current-year twigs, (C) reproductive twigs, (D) vegetative twigs.


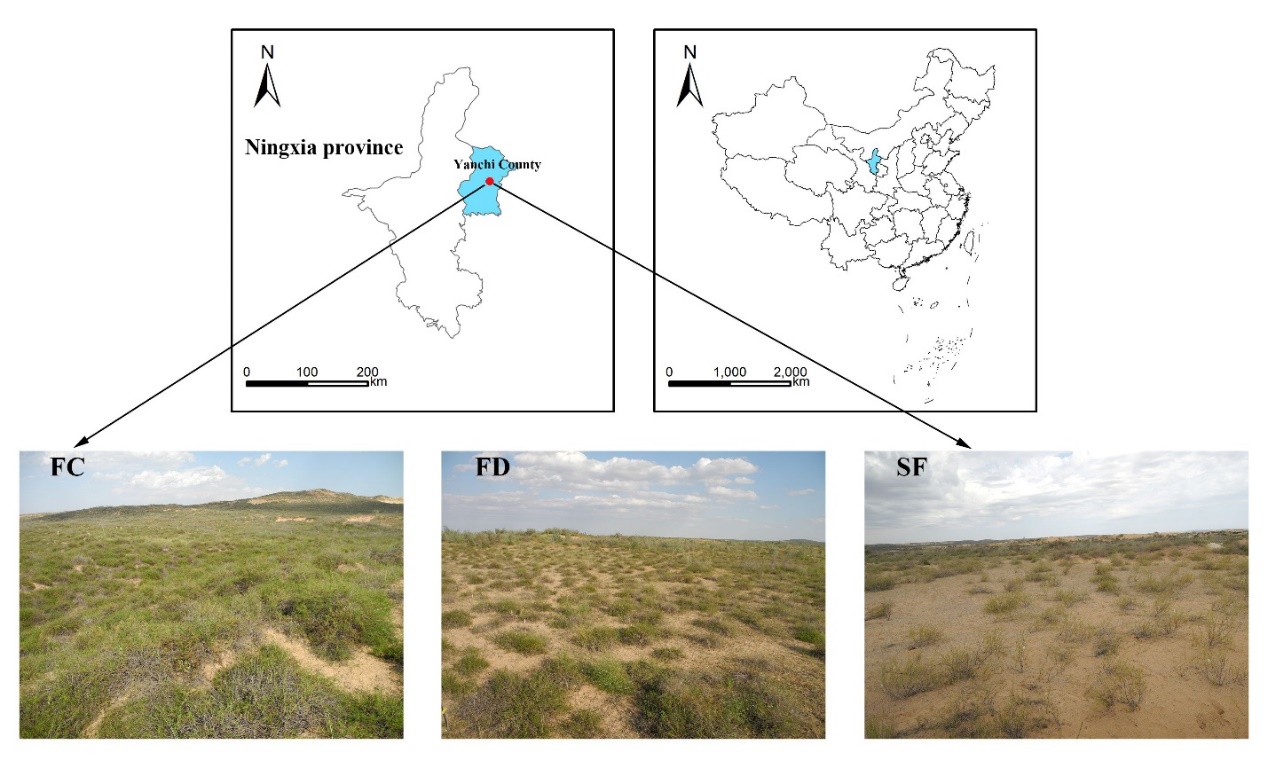
**Figure S2.** Map of the study site, and photographs of the three distinct habitats: FC (fixed dunes covered with biological soil crusts), FD (fixed dunes) and SF (semi-fixed dunes).


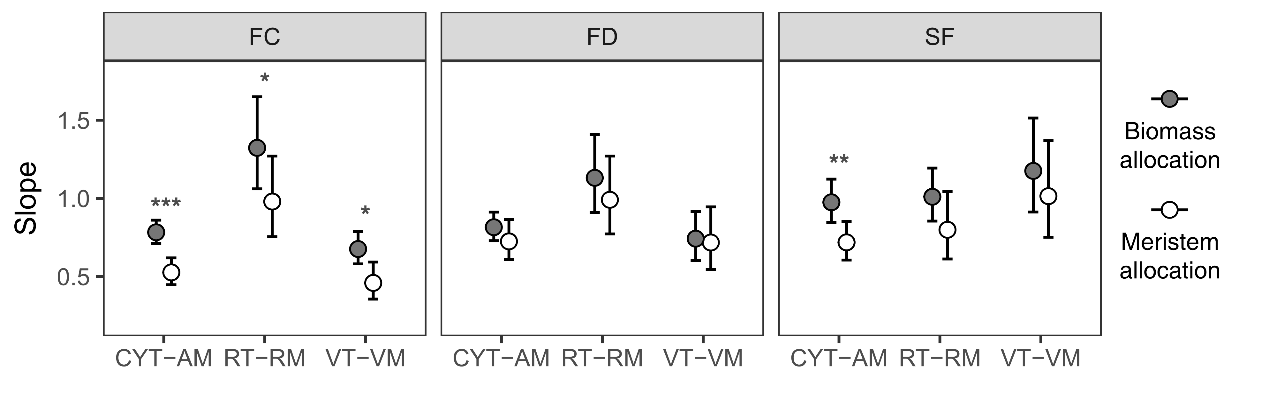


**Figure S3.** Allometric slope comparisons between size-dependent biomass allocation and size-dependent meristem allocation of *Artemisia ordosica* across habitats. For instance, CYT−AM indicates the allometric slope comparison between the CYT biomass–plant biomass relationship and the AM number–plant biomass relationship. * *P* < 0.05, ** *P* < 0.01, *** *P* < 0.001. Abbreviations: FC, fixed dunes with crusts; FD, fixed dunes; SF, semi-fixed dunes; CYT, current-year twig; RT, reproductive twig; VT, vegetative twig; AM, active meristem; RM, reproductive meristem; VM, vegetative meristem.
